# Maternal death at 28 weeks gestation due to vascular rupture of placenta accreta

**DOI:** 10.1016/j.xagr.2025.100562

**Published:** 2025-08-27

**Authors:** Khaoula Magdoud, Ons Hmandi, Sana Menjli, Ines Ben Hassen, Imen Labidi, Eya Azouz, Bilel Arfaoui, Hassine Saber Abouda

**Affiliations:** 1 Faculty of Medicine of Tunis, University of Tunis El Manar, Tunis, Tunisia (Magdoud, Hmandi, Azouz, and Abouda); 2 Emergency Department, Maternity and Neonatology Center of Tunis, Tunis, Tunisia (Magdoud and Laabidi); 3 Legal Medicine Department, Charles Nicolle Hospital, Tunis, Tunisia (Hmandi); 4 Department C, Maternity and Neonatology Center of Tunis, Tunis, Tunisia (Menjli, Hassen, Arfaoui, and Abouda); 5 Radiology Department, Rabta Hospital, Tunis, Tunisia (Azouz)

**Keywords:** autopsy, maternal death, placenta accreta, second pregnancy trimester

## Abstract

Placenta accreta spectrum (PAS) is becoming increasingly common and is associated with significant morbidity and mortality. Prenatal diagnosis and timely referral to centers have been shown to improve outcomes. We present a case of maternal death at 28 weeks of gestation due to massive internal bleeding caused by the rupture of abnormal blood vessels from placenta accreta. The diagnosis of PAS was suspected by morphological ultrasound at 23 weeks of gestation. Expectant management was decided for this patient since she had no children. The patient was transported to the emergency room at 28 weeks of gestation after having a sudden loss of consciousness without other signs (no pelvic pain, no metrorrhagia). After preliminary assessment, maternal death was noted. Hemoperitoneum associated with a placental vascular rupture was found at the autopsy. In the absence of a therapeutic consensus, this case highlights the challenges in managing pregnant women with suspected PAS disorders in the second trimester.

## Introduction

Placenta accreta spectrum (PAS) disorders are the result of abnormal attachment or invasion of the placental chorionic villi directly to the underlying myometrium.^1^ Today, uterine scar secondary to cesarean delivery represents the main risk factor of placenta accreta.^1^ PAS disorders are an important cause of maternal morbidity and mortality, usually due to significant obstetric bleeding.^2^^,^^3^ Ultrasound is the primary screening and diagnostic modality of PAS.^4^ According to recent guidelines, scheduled non emergent cesarean hysterectomy is the preferred course of treatment for the great majority of patients with PAS disorders, yet the optimal timing remains unclear.^5^ In case of PAS diagnosis in the second trimester of pregnancy, expectant management remains possible.^6^

In this case report, we present a cataclysmic rupture of the vessels of a placenta accreta without uterine rupture at 28 weeks of pregnancy, causing maternal death.

## Clinical presentation

We report a case of a 41-year-old patient, gravida 6, para 2, with history of four early pregnancy losses requiring surgical evacuation and preterm cesarean section due to fetal distress. The patient has no living children. During this pregnancy, she was on low-dose aspirin and low-molecular-weight heparin and had been diagnosed with gestational diabetes and pregnancy-induced hypertension.

**Figure 1:**
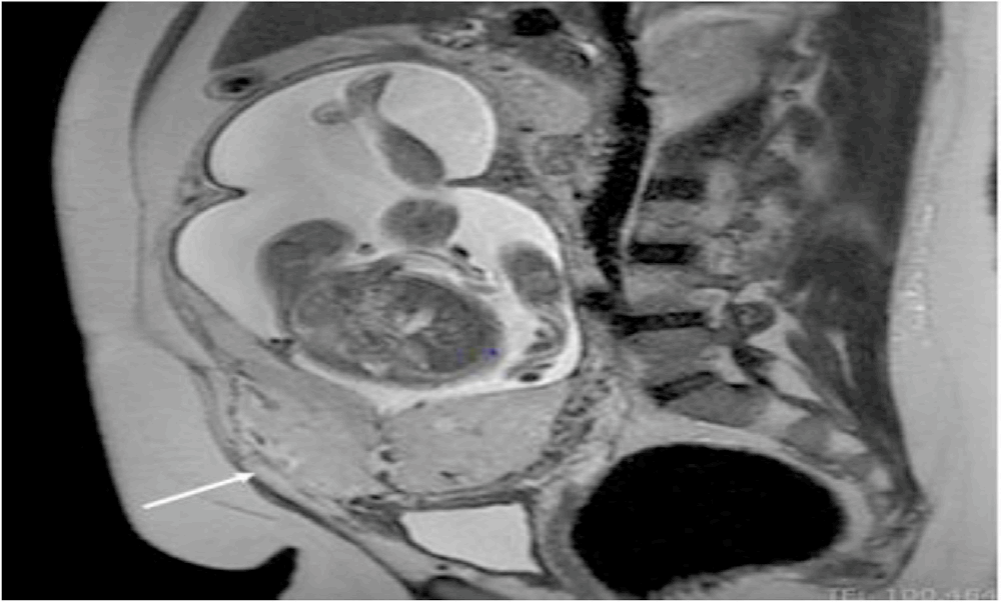
MRI sagittal T2-weighted image showing a completely covering placenta previa, loss of continuity in the placental-myometrial interface at the isthmic level with anterior bulging (arrow).

Ultrasound findings at 23 weeks showed a complete anterior placenta previa with signs of placenta accrete. In the anterior uterine wall, we noted placental lacunae, subplacental hypervascularity, and bridging vessels. A placental MRI performed at 27 weeks confirmed completely covering placenta previa, loss of continuity in the placental-myometrial interface at the isthmic level with anterior bulging (Figure 1). According to the FIGO classification, placenta accreta was classified as grade 2 (placenta increta).^7^

Magdoud. Maternal death at 28 weeks gestation due to vascular rupture. AJOG Glob Rep 2025.

Expectant management was decided after the discussion with the patient and her husband. The couple was informed of the risks, including uterine rupture and emergency caesarean section.

At 28 weeks of gestation, the patient was brought to the emergency department with sudden loss of consciousness without other signs (no pelvic pain, no metrorrhagia). Upon arrival, she was unresponsive, not breathing, pulseless, with cold extremities and bilateral mydriasis. There were no signs of external trauma. Resuscitation was attempted for 20 minutes but was unsuccessful, and death was declared. A bedside ultrasound showed moderate internal bleeding, an intrauterine fetus with confirmed fetal death, and a normal amniotic fluid volume. In the absence of obvious causes of death, including uterine rupture, an autopsy was indicated.

**Figure 2:**
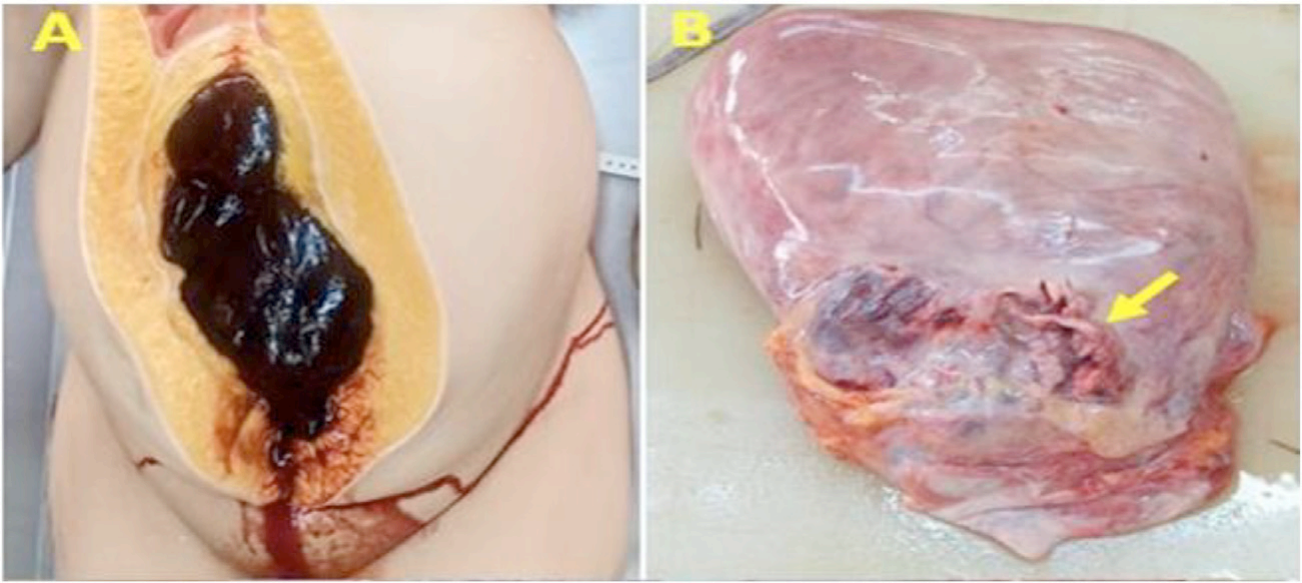
Autopsy findings. (A) Large hemoperitoneum with clotted blood. (B) Rupture of blood vessels in placenta percreta (yellow arrow) at the posterior uterine wall.

The autopsy revealed significant internal hemorrhage; the anterior uterine wall was intact. At the posterior wall of the uterus in the isthmic part, there was a rupture of abnormal blood vessels from a placenta percreta. The fetus was in the uterus with normal amniotic fluid (Figure 2).

Magdoud. Maternal death at 28 weeks gestation due to vascular rupture. AJOG Glob Rep 2025.

## Discussion

PAS disorders refer to the penetration of trophoblastic tissues through the decidua basalis into the uterine myometrium, uterine serosa, or beyond, extending to the parametrium or the adjacent pelvic organs.^8^

The risk factors of PAS disorders are advanced maternal age, multiparity, use of assisted reproductive technologies, prior uterine surgery (curettage, endometrial ablation, myomectomy), previous cesarean sections, and placenta previa.^7^ Today, uterine scar secondary to cesarean delivery represents the main risk factor of placenta accreta, and the risk is correlated with the number of cesarean section.^9^

The incidence of PAS has increased over the past several decades in conjunction with the rate of cesarean deliveries.^10^ PAS disorders complicated one in 313 of all deliveries in 2017 in the United States.^10^ With 52% of cases requiring peripartum hysterectomy, PAS diseases are an important cause of maternal morbidity and mortality, usually due to significant obstetric bleeding.^2^^,^^3^ the severity of PAS disorders range from superficial adhesion of placental (placenta accreta) to varying degrees of myometrial invasion (placenta increta or placenta percreta).^7^

Ultrasound is the primary screening and diagnostic modality of PAS.^3^ The most common signs are myometrial thinning (less than 1 mm), placental lacunae, subplacental hypervascularity, bridging vessels, and a loss of the normal hypoechoic zone, between the placenta and myometrium.^3^ The meta-analysis of Pagani et al^11^ showed ultrasound sensitivity for identification of placenta accreta, placenta increta, and placenta percreta of 91%, 93%, and 81% and specificity of 97%, 98%, and 99%, respectively.

However, in some cases, placental MRI should be indicated. The main interest of placental MRI is the more in-depth assessment of myoinvasion, because MRI accurately describes the depth and topography of invasion. Thus, it guides the surgical technique and avoids transplacental incision.^12^ the second indication for MRI is the posterior location of the placenta. The optimal time for MRI evaluation of PAS disorders is 28 to 32 weeks gestation.^12^

The common signs of PAS disorders in MRI are intraplacental T2-dark bands, placental or uterine bulge, myometrial thinning, bladder wall interruption, focal exophytic mass, loss of T2-hypointense retroplacental line, and abnormal vascularization of the placental bed.^12^

According to the American College of Obstetricians and Gynecologists and the Society for Maternal-Fetal Medicine, scheduled non emergent cesarean hysterectomy is the preferred course of treatment for the great majority of patients with PAS disorders.^5^ Prophylactic cesarean section is recommended between 34 0/7 and 35 6/7 weeks of gestation to avoid the risk of emergency cesarean delivery.^5^ Indeed, delivery in the event of PAS disorders in an emergency context will increase maternal mortality and morbidity.^13^ However, expectant management until 36 to 37 weeks of gestation is not associated with an increase in maternal morbidity and mortality but it improves neonatal prognosis.^14^

Early diagnosis of PA in the second trimester represents a complex obstetric situation, and there is no consensual management of pregnancy.

The decision will depend on the patient’s wishes, the degree of myometrial invasion, the absence of comorbidities, and the experience of the surgical team.

Some teams opt for termination of the pregnancy with radical treatment (hysterectomy) or conservative treatment.^15^ This attitude is explained by a higher risk of uterine rupture reported in several case series.^16^

Our case was unique compared to the reported cases, since there was no solution of continuity of the uterine wall, but only an isolated vascular rupture. Which explains a normal amniotic fluid on ultrasound. The fetal death was secondary to the maternal hemorrhagic shock and not the uterine rupture.

Patients with placenta previa status and PAS requiring an induced abortion on second-trimester present a high risk of massive hemorrhage both during delivery and operation. There are few reports on these cases, so it is still unclear which treatment is most appropriate. An effectively tailored surgical approach is urgently needed to account for the serious life-threatening complications.^17^

Thus, the challenge of second-trimester management presents a delicate balance between maternal health, neonatal outcomes, patient autonomy, and morbidity. Further studies should be conducted to determine whether differing techniques for second-trimester PAS. Multidisciplinary teams should include providers that can manage continuing pregnancies for those choosing expectant management and providers that can provide second-trimester abortion care and active management, if the patient decides for active management.^18^

Mid-trimester PAS induction is not commonly performed; it is necessary to make individualized treatment decisions based on the disease characteristics and wishes of the patients.^19^

## Conclusion

Rupture of the vessels of placenta percreta during pregnancy is an exceptional but sometimes fatal complication. Therefore, expectant management in cases of PAS disorders diagnosis in the second trimester should be reviewed.

## CRediT authorship contribution statement

**Khaoula Magdoud:** Writing – review & editing, Writing – original draft, Data curation, Conceptualization. **Ons Hmandi:** Writing – review & editing, Data curation. **Sana Menjli:** Project administration. **Ines Ben Hassen:** Writing – review & editing. **Imen Labidi:** Writing – review & editing. **Eya Azouz:** Supervision, Data curation. **Bilel Arfaoui:** Validation, Supervision. **Hassine Saber Abouda:** Writing – review & editing, Supervision.
